# Impact of the Konio pathway in the thalamocortical visual system: a modeling study

**DOI:** 10.1186/1471-2202-14-S1-P6

**Published:** 2013-07-08

**Authors:** Carlos Carvajal, Thierry Viéville, Frédéric Alexandre

**Affiliations:** 1 Inria, Mnemosyne Team, Bordeaux Sud-Ouest Research Center, 33400 Talence, France; 2 LaBRI, Université de Bordeaux, Institut des Maladies Neurodégénératives, 33000 Bordeaux, France; 3 Université de Lorraine, LORIA UMR 7503, 54600 Villers-lès-Nancy, France

## Background

In the early visual system, regarding the detection of a visual event, motion information is pre-processed in the Magnocellular pathway, while it has been shown that the Koniocellular pathway [1] also plays an important role, providing a global analysis about such a kind of information processing. However, the functional interplay between these two parallel pathways remains partially understood. Previous works have attacked this question by studying the signals produced by the corresponding ganglion cells [1] and their elaboration at further steps [2], rather than proposing to model the underlying mechanisms at a mesoscopic level, i.e., focusing on the functional aspects of such dual processing. Neurobiological studies dedicated to the thalamocortical stage of the early visual system provide knowledge on particular characteristics of the system, namely: 1) the variety of cell types in the retina [2], inducing different pathways, 2) the variety of thalamocortical projections through focused vs diffuse efferences to the cortex [3], from core vs matrix (specific vs non-specific) thalamic nuclei, and 3) the variety of kinds of connectivity between thalamic, cortical and collicular areas (i.e., feedforward, feedback, shortcuts, driver and modulator information flows [4]).

## Methods

To figure out the impact of these multi-scale characteristics, we propose here a systemic approach at the structure level. To this end, we have developed a reduced bio-inspired distributed asynchronous model of the primitive mammal visual system, considering only motion event detection. This computational model is fed with natural image sequences, and is implemented as a large size distributed calculation [5] with thousands of computation units per structure.

## Results

Thanks to the dual analysis integrating local and larger image cues, we test the system for the detection of specific dynamical patterns (which could be interpreted as, e.g., threats or targets). Our simulations aim at showing that these multi-scale interactions help improving the speed and/or quality of such critical tasks, including target selection and tracking. We expect this approach to propose an innovative answer to the interplay issues quoted here, generalizable to other visuo-motor functions. This also provides a platform that could be used as a testbed for new hypotheses. Further information flows to be included could correspond to the Parvocellular pathway (and its related functions). Other structures such as the pulvinar, or higher cortical areas, would also allow us to explore even more developed mechanisms and behaviors [3,6].
